# Deep learning for automatic calcium detection in echocardiography

**DOI:** 10.1186/s13040-024-00381-1

**Published:** 2024-08-28

**Authors:** Luís B. Elvas, Sara Gomes, João C. Ferreira, Luís Brás Rosário, Tomás Brandão

**Affiliations:** 1 Department of Logistics, Molde University College, Molde, 6410 Norway; 2 Instituto Universitário de Lisboa (ISCTE-IUL), ISTAR, Av. das Forças Armadas, Lisboa, 1649-026 Portugal; 3 Inov Inesc Inovação—Instituto de Novas Tecnologias, Lisbon, 1000-029 Portugal; 4 Faculty of Medicine, Lisbon University, Hospital Santa Maria/CHULN, Centro Cardiovascular da Universidade de Lisboa (CCUL@RISE), Lisbon, 1649-028 Portugal

**Keywords:** Cardiovascular diseases, Cardiac diseases, Aortic stenosis, Aortic sclerosis, Aortic calcification, Diagnosis, Echocardiography, Data-driven tool, Deep learning (DL), Convolutional neural networks (CNNs), Object detector, Image classification

## Abstract

Cardiovascular diseases are the main cause of death in the world and cardiovascular imaging techniques are the mainstay of noninvasive diagnosis. Aortic stenosis is a lethal cardiac disease preceded by aortic valve calcification for several years. Data-driven tools developed with Deep Learning (DL) algorithms can process and categorize medical images data, providing fast diagnoses with considered reliability, to improve healthcare effectiveness. A systematic review of DL applications on medical images for pathologic calcium detection concluded that there are established techniques in this field, using primarily CT scans, at the expense of radiation exposure. Echocardiography is an unexplored alternative to detect calcium, but still needs technological developments. In this article, a fully automated method based on Convolutional Neural Networks (CNNs) was developed to detect Aortic Calcification in Echocardiography images, consisting of two essential processes: (1) an object detector to locate aortic valve – achieving 95% of precision and 100% of recall; and (2) a classifier to identify calcium structures in the valve – which achieved 92% of precision and 100% of recall. The outcome of this work is the possibility of automation of the detection with Echocardiography of Aortic Valve Calcification, a lethal and prevalent disease.

## Introduction

In 2019, cardiovascular disorders accounted for one-third of all global fatalities [1], marking a concerning increase over the years [2]. The most frequent heart valve disease is Aortic Stenosis (AS), which is a lethal disease and a major cause of cardiovascular death [3]. Several mechanisms lead to the calcification of the aortic valve leaflets which is commonly thought to be the cause of Aortic Valve Sclerosis (AVS) or Aortic Valve Calcification (AVC) [4]. AVC is a relatively slow progressing disease with several years lapse between the first lesions and severe AVS, therefore requiring follow up exams during a patient lifetime.

Medical imaging modalities, including Echocardiography and Computed Tomography (CT) can identify calcium in anatomic structures due to the specific reflective and absorptive properties of calcium compounds [5]. CT has a high image quality and provides a great predictive capability, but its repetitive use can be hazardous, due to ionizing radiation exposure [6]. On the other hand, echocardiography is the first-line method for diagnosing and monitoring the evolution of Aortic Stenosis, according to the European Society of Cardiology [7], due to its portability, low cost and, especially, zero radiation exposure [7].

Numerous studies, as referenced in [8], have focused on the application of Artificial Intelligence for automated calcium measurement on CT scans. However, a significant gap persists in exploring the potential of echocardiography-based automatic calcium detection [9]. CNNs demonstrate an aptitude for capturing spatial relationships within echocardiographic images, their ability to discern subtle patterns indicative of aortic valve calcification arises from training on relevant data and the use of appropriate loss functions [10]. Moreover, the end-to-end learning capability of DL models, as highlighted in [8], allows for the direct acquisition of both low-level and high-level representations from raw imaging data, eliminating the need for labor-intensive manual feature engineering. Transfer learning further enhances such approaches, enabling the fine-tuning of pre-trained models on expansive datasets. This proves especially beneficial in the medical imaging domain where labeled datasets for echocardiography are limited. By adapting models trained on broader datasets to our specific task, we harness the general image features that are essential for identifying aortic valve calcification patterns. CNNs have demonstrated considerable success in tasks such as image segmentation and classification [11–16], emphasising their efficacy for our challenge.

In our previous literature review [20] 82 references, highlighting the role of deep learning, calcium scoring, and CT scans in cardiac imaging. Some approaches demonstrated superior results, emphasizing AI’s potential in improving healthcare efficiency. Despite radiation risks, CT scans remain the primary choice for automated DL applications, but ongoing developments suggest an emerging viability of fully automated echocardiography methods, promising enhanced patient monitoring and reduced physician workload, while annulling radiation exposure compared to CT scans. In [9], a semi-automatic model for calcium detection in echocardiographic images suggested potential for a more efficient fully automatic solution, but for that purpose automatic valve recognition and calcium identification methods are necessary.

This being said, the primary goal of this study is to automate the detection of AVC in patients by identifying calcium deposits in the aortic valve using echocardiographic data, contrasting with solutions discussed in the literature review. To achieve this objective, this work was divided into two distinct problems: (1) the development of an object detection algorithm for identifying the aortic valve and (2) the creation of a classification algorithm to determine the presence of calcium within the valve. DL techniques were tried, using CNNs, and a manual classification approach was also followed, for results comparison. Recognizing the scarcity of extensive datasets — a challenge highlighted by the European Parliamentary Research Service [17] — our study focuses an effective CNN model using a small dataset, specifically in the context of echocardiography. We tackle this limitation by introducing data augmentation techniques and leveraging computer vision methods. Despite these constraints, our approach yields promising results, showcasing the potential of AI in cardiac diagnostics related to Aortic Calcification and Echocardiography Imaging. In addition to addressing data scarcity, our study has broader implications. By automating the detection of AVC and identifying calcium deposits in the aortic valve, we not only contribute to cardiac diagnostics but also alleviate the burden on clinicians. Consider this: in a typical clinic, where 30 echocardiographies are performed daily, each taking approximately 10 min for manual analysis, our approach could save up to 5 h per day. Moreover, for future studies or large-scale patient screenings, manual effort would be impractical to quantify. Our method is expected not only to accelerate the process but also to optimize it, given that it is fully automated.

The structure of this article aligns with the Cross Industry Standard Process for Data Mining (CRISP-DM) methodology, widely recognized as the most used framework for data mining and analytics [18]. The CRISP-DM methodology comprises six phases, with (1) Business Understanding and (2) Data Understanding represented in sub-chapter “2.1. Business and Data Understanding”, (3) Data Preparation encompassing sub-chapters “2.2. Pre-processing”, “2.3. Annotations”, and “2.4 Data Augmentation”, (4) Modeling found in chapter “3. Modeling”, (5) Results detailed in “3.1.3 Experimental Procedures results” and “3.1.4 Experimental Procedures”, and finally, (6) Deployment is elucidated in this study.

## Materials and methods

### Business and data understanding

The data selection process was performed under the rules of a pre-signed confidentiality agreement to ensure compliance with data protection regulations while striving to create an appropriate dataset for the application of DL models. A confidentiality agreement was executed through collaboration with Affidea IMI Lisbon - República Clinic (Portugal) to safeguard patients’ data. To comply with ethical guidelines, informed consent was obtained from participants, following principles outlined in the Declaration of Helsinki and the Oviedo Convention [19]. Additionally, all members with data access adhered to GDPR regulations, safeguarding sensitive information, specifying authorized personnel, defining data retention periods, establishing data disposal procedures, and preventing unauthorized utilization in other research contexts without explicit consent.

Under the guidance of a cardiologist from the Affidea Clinic, actively involved in all stages, consecutive 2D echocardiography exams with a probe of 3.5 MHz were chosen exclusively from consenting patients, and saved in Dicom format [20].

Each of these files consists of two components: a set of images captured during the exam using sonography, and the associated metadata containing the patient’s information. During the saving process, files were assigned anonymous names, such as “STUDY_000”, “STUDY_001”, and so forth, to ensure compliance with the general data protection regulations (GDPR) and scientific integrity. Additionally, any information data that could identify the patients was deleted from the images.

A total of 70 exams, each one containing a set of ultrasound images, extracted from the device model LOGIQ S8 [21], were copied, pre-processed, filtered and utilized in modeling experiments.

The 41 patients in this study have an average age of 69.31 years with a standard deviation of 12.03. The maximum age observed is 86, and the minimum is 52. The gender distribution is 35.7% male and 64.3% female.

### Pre-processing

From the initial selection of echocardiograms, a small subset unrelated to heart conditions was excluded (22 images), with each medical examination comprising approximately ten images. The initial pre-processing involved extracting images from the Dicom files, storing them with the examination ID in their names for easy identification, since in the anonymization process all the personal data was removed.

**Fig. 1:**
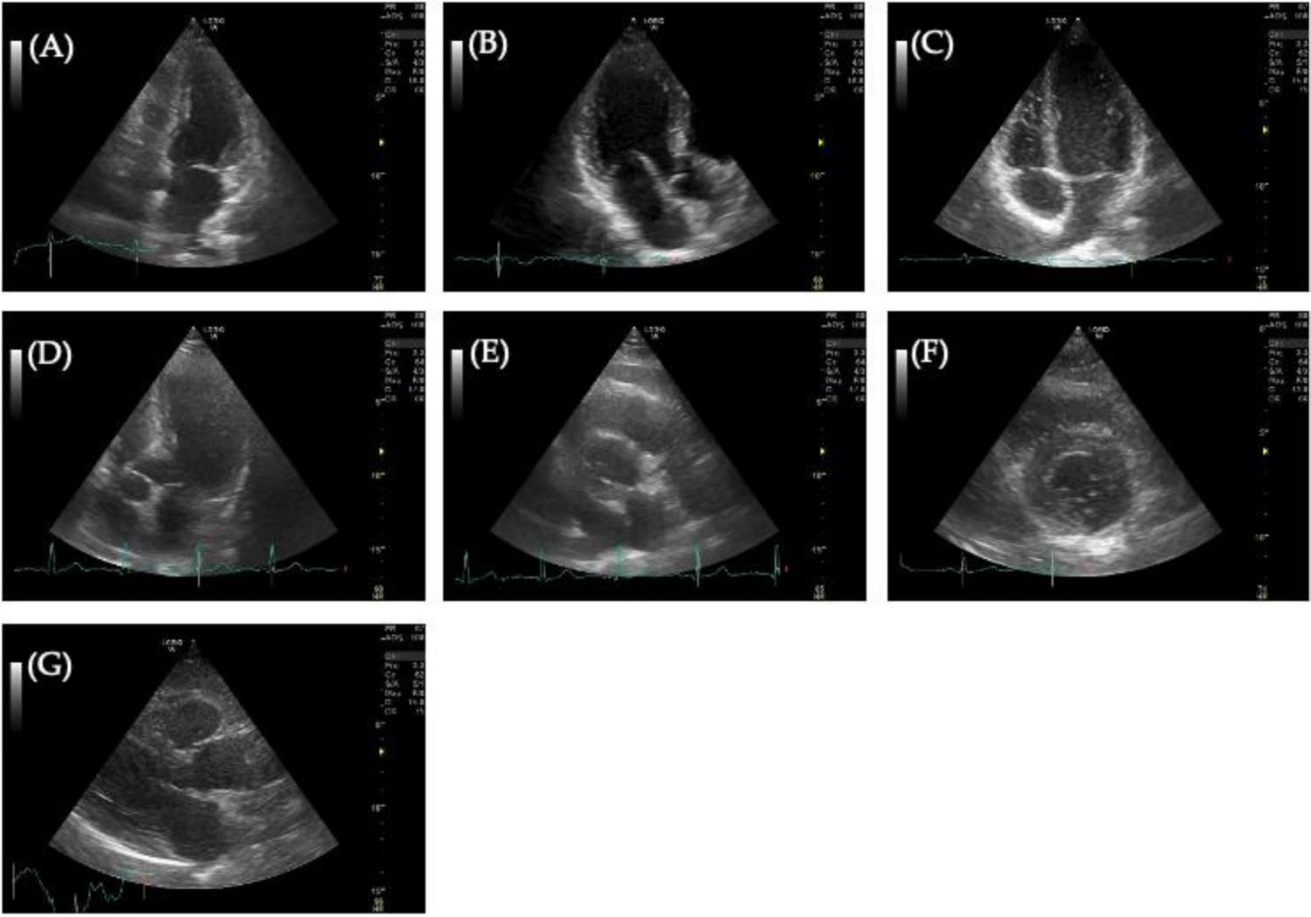
Different types of heart view on Echocardiography: (**A**) Apical 2-chamber view; (**B**) Apical 3-chamber view; (**C**) Apical 4-chamber view; (**D**) Apical 5-chamber view; (**E**) Parasternal short-axis view; (**F**) Parasternal short-axis view of the left ventricle; (**G**) Parasternal long-axis view

During an echocardiography examination, the physician captures images of the heart from pre specified views, as illustrated in Fig. 1.

For the designated purpose, it was determined to exclusively employ the parasternal short-axis views (Fig. 1 - E) during diastole, as these images feature the aortic valve, which constitutes the target of identification. Following the selection of only this specific view, a dataset comprising 61 images from 41 patients where 36% of which suffer from AVC has been created.

**Fig. 2:**
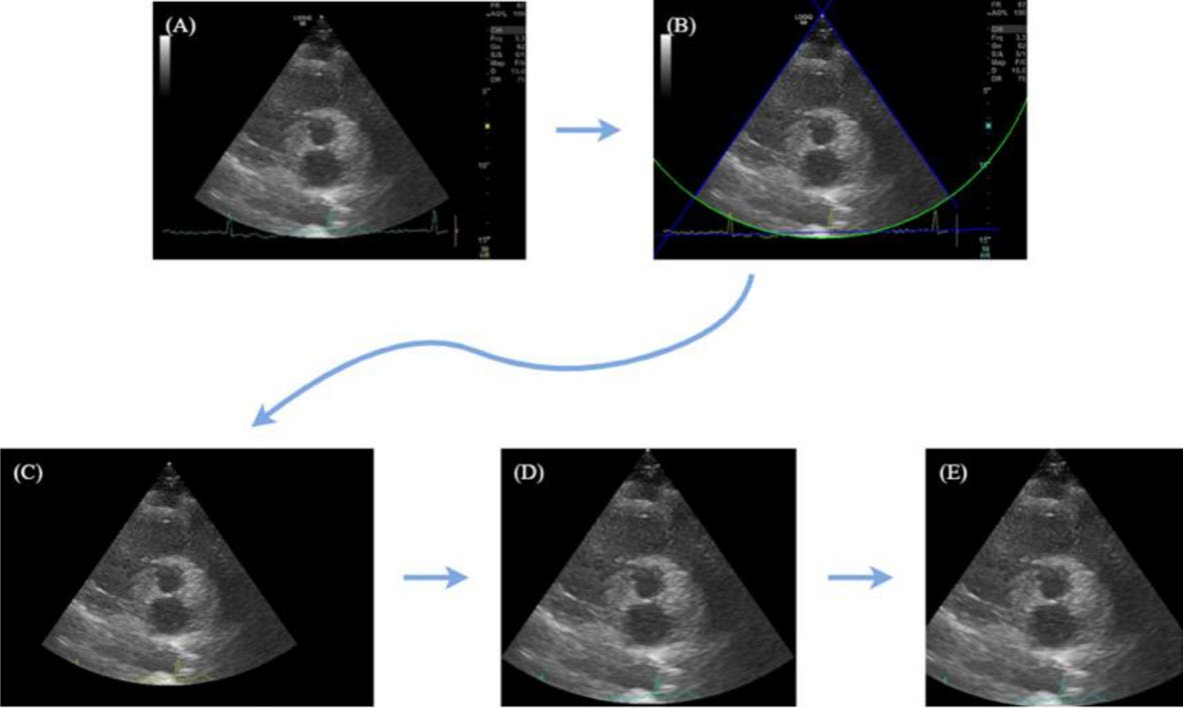
Image transformation process

The subsequent stage, illustrated in Fig. 2, consisted of extracting and normalizing the dimensions of interest regions, using Python image processing tools. This process aimed to simplify the images by eliminating useless details that are automatically inserted into the images by the capturing device (which would otherwise be fed into the Neural Networks as noise).

Starting with the original echocardiography image (A), our region of interest is within the cone and our goal is to identify and crop the image accordingly. In (B), contours are identified using OpenCV library functions [22] that detect straight lines and circles. Moving to (C), we select our region of interest by creating a binary mask within the three lines. In (D), edges are cropped to minimize unnecessary black areas, and in (E), all images are manually cropped to standardized dimensions of 640 × 640 pixels. This standardization aims to prevent image deformation and feature loss. DL models require specific input shapes, and automatic resizing could impact results by changing the aspect ratio. We chose the dimension 640 × 640 pixels to match the input shape of the object detection model used in experiments (YOLOv5 [23]).

### Annotations

**Fig. 3:**
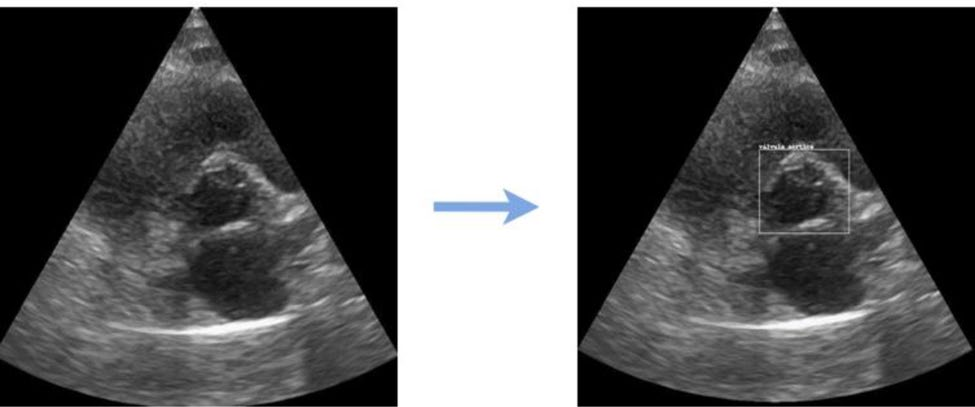
Image annotation process

In order to address the first goal, training an object detector is necessary, requiring annotated images where objects are identified by bounding boxes indicating their locations. Using MAXQDA [24], a data analysis software, bounding boxes were manually drawn for all 61 images selected for the modeling phase, saving annotations as rectangle coordinates in a .csv file. Annotated images enable the model to extract crucial features during training, and its performance is evaluated in the testing phase by comparing predictions with real object locations, completing this crucial step for achieving the first goal. Figure 3 illustrates this process for one example image.

### Data augmentation

As the minimum dataset size to perform an object detector is approximately 150–500 images [25], the dataset obtained with 61 images is too small to achieve satisfactory results in the modeling phase. A commonly employed strategy to compensate for limited data availability is the application of image augmentation, which involves employing image transformation techniques to generate new images based on variations of the original ones, thereby expanding the dataset and introducing diversity for the model to learn from [26].

**Fig. 4:**
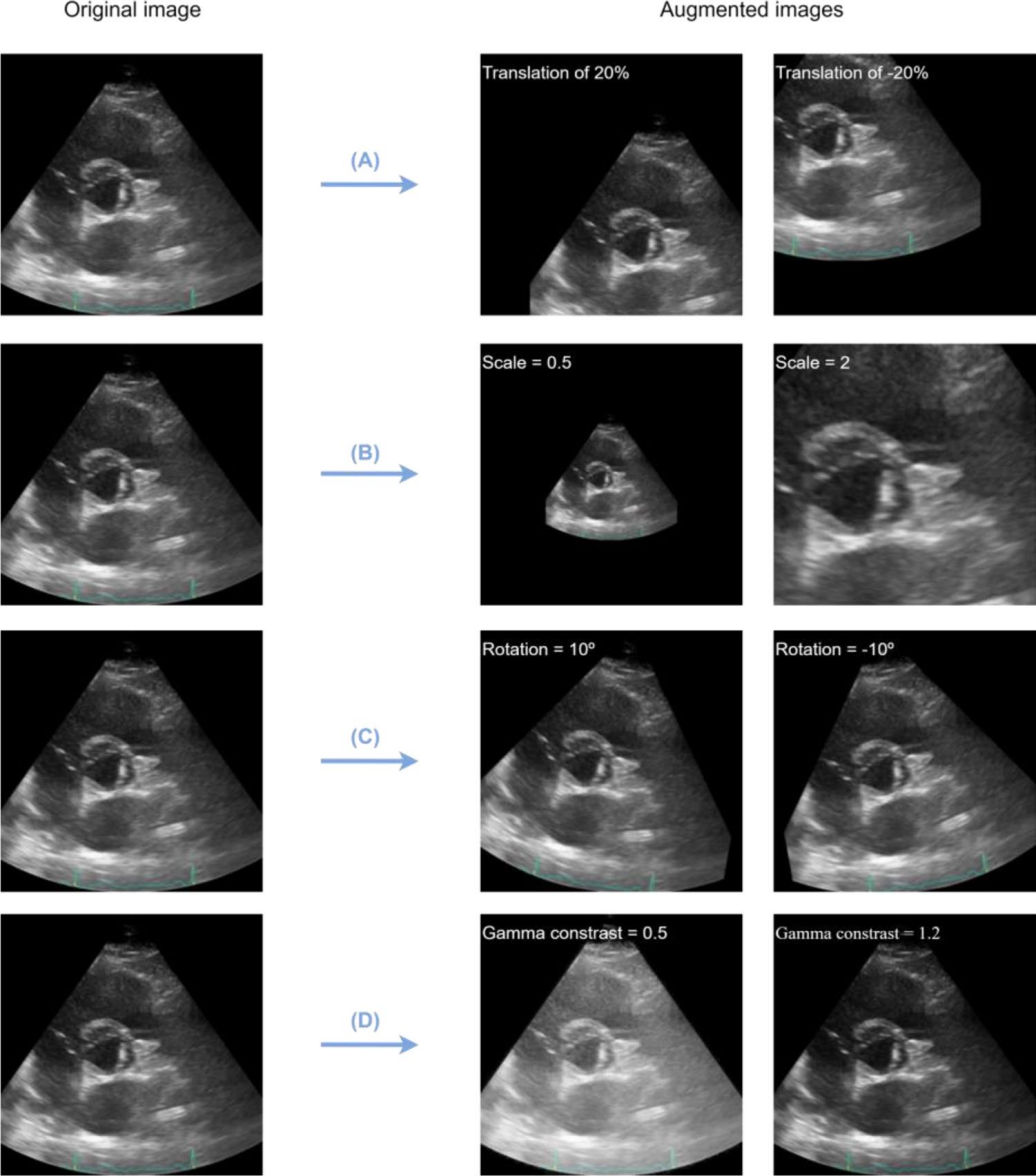
Data augmentation process. (**A**) Translation; (**B**) Zoom; (**C**) Rotation; (**D**) Gama contrast

In this case, 4 types of data augmentation techniques were applied, to create a larger dataset before initializing the model train. The methods used were translation, rotation, zoom and gamma contrast, using a Python library called *imgaug* [27], which also transforms annotations directly, generating the corresponding object location in the augmented image version. In Fig. 4, the images obtained from an original one by using each one of these techniques are illustrated.

A. Translation. Translation percentage set to 0.2 and − 0.2.
B. Zoom. Image scale set to 0.5 and 2.
C. Rotation. Rotation angle set to 10º and − 10º.
D. Gamma contrast. Contrast set to 0.5 and 1.2.

The rotation threshold was chosen according to the medical specialist’s advice, justified by the exam procedure, where ultrasound position may vary about generally 10 degrees clockwise or counterclockwise from technician to technician. The other thresholds were chosen considering that the valve must be entirely inside the image after the transformation.

## Modeling

**Fig. 5:**
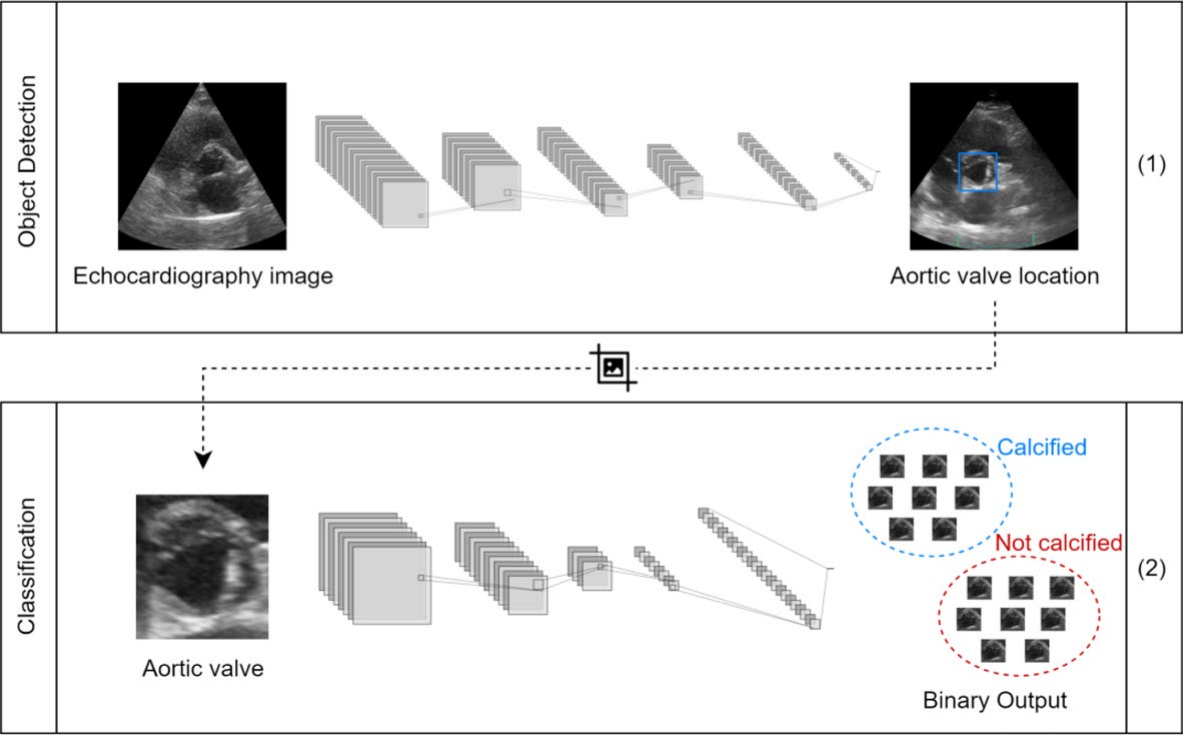
Framework for aortic valve detection and Aortic Sclerosis classification

In the modeling phase, the main purpose is to build a framework for detection of AVC in echocardiography images, by detecting calcium structures in aortic valve. To achieve the proposed goal, the development of two separate algorithms was necessary, as illustrated in Fig. 5: (1) an object detector whose goal is to detect the aortic valve in each image, after training it with the created dataset; (2) a ML-based algorithm that classifies the image of the aortic valve according to the presence of calcium. For the second algorithm, a comparison between a DL approach and a heuristic classification was done, showing advantage on the use of DL models.

The simplest approach for this problem would use only the classification part, training the classifier with the entire original images. It would simplify the developments, but the expected results were very low, considering the small dataset size. Adding an object detector before the classification step, the classifier will be trained using only the area of interest, which is aortic valve. This makes it easier for the model to extract key features and to perform better.

### Aortic valve detection

For the first algorithm, being the goal to automatize the identification of an object from an image (in this case the object is aortic valve), it was necessary to train an object detector. This CV technique consists of identifying objects within an image, by means of providing the bounding boxes around the detected objects.

#### Object detector modeling

There are two main ways to train an object detector using DL [28]: (1) using Transfer Learning to apply a pre-trained model; (2) training a Neural Network from scratch, using a custom dataset.

1. Transfer Learning can be a faster method and achieve better results, as it uses a model that has already been trained with a huge quantity of data.
2. Training from scratch requires a large amount of labeled data and typically requires more time to be trained.

**Fig. 6:**
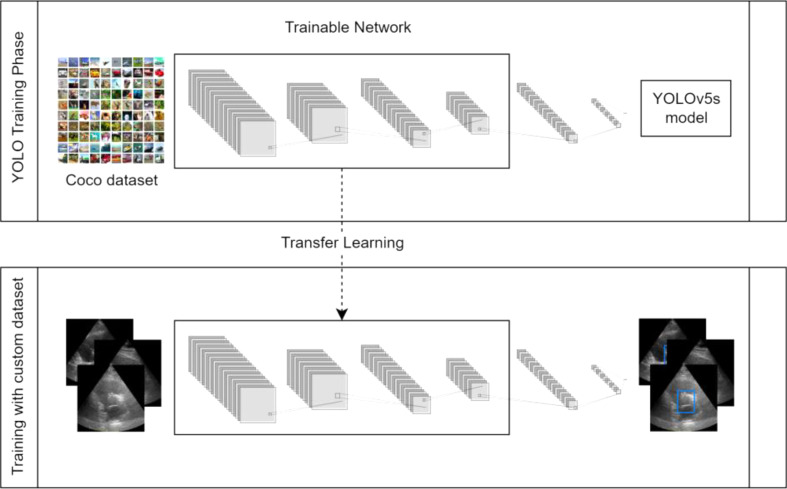
Object detector with Transfer Learning for aortic valve detection

The approach (1) was followed in this work, motivated by the lack of available images. The obtained dataset with 61 images would not be enough to build and train a model from scratch. As represented in Fig. 6, a pre-trained model, previously trained with a large set of data called, was selected and its weights were used to initialize the NN. Then the next layers were trained using custom data – the echocardiography dataset with 61 images.

There are many useful libraries to work with CV, specifically with object detection, such as TensorFlow [29], Keras [30], PyTorch [31] and OpenCV [22]. To select a pre-trained model, it is important to consider the size and complexity of the selected dataset. From the variety of available models, YOLO’s version 5 (YOLOv5) was chosen for demonstrating consistent performance, representing a turning point for YOLO’s model versions, as it is the first one built on the PyTorch framework [23]. Since working with a small set of images, the YOLOv5s variation – which is designed for small datasets – was selected in this case.

#### Experimental procedures

In the modeling phase, the object detector was trained with the constructed dataset, containing 61 images. The following parameters were defined when creating the NN:

- Input shape à (640, 640, 3).
- Number of batches à 32.
- Number of epochs à 75.
- Dataset split à 80% for train + 10% for validation + 10% for test.

The images resulting from 4 types of data augmentation techniques, described in Subsection 2.4., were gradually included, to train the object detector with a larger dataset. Each data augmentation technique was included individually (Experiments 2 to 5), then a dataset using all the techniques simultaneously was used (Experiment 6), and finally a model using the original images and the data augmentation images resulting from the two techniques that had the best individually performances – translations and rotations – was trained (Experiment 7).

All the results are summarized in Table 1.

**Table 1:** Object detection results

Experiment	Dataset size	Precision	Recall	F1-Score	Description
1	61	1.00	0.26	0.44	Original dataset
2	183	1.00	0.81	0.89	Data Augmentation - zoom
3	183	1.00	0.95	0.98	Data Augmentation - translation
4	183	0.95	1.00	0.98	Data Augmentation - rotation
5	183	0.95	0.90	0.93	Data Augmentation – gamma contrast
6	549	0.91	0.97	0.94	Data Augmentation - zoom, translation, rotation and gamma contrast
7	305	0.93	1.00	0.97	Data Augmentation - translation and rotation

The first experiment, without data augmentation, achieved a low performance. Although having a 100% precision, it registered only 26% of recall, which means that all the model predictions were correct, but it could not detect all the actual objects (only 26%).

By including the image sets obtained from each data augmentation technique, the results increased significantly, with highlight for translation and rotation (experiments 3 and 4). The use of translation for enlarging the dataset increased the precision to 100% and recall to 95%, meaning that all the model predictions were correct, and almost all the actual locations of the valve were identified (only 5% were not found). Rotation, on the other hand, achieved 95% of precision and 100% of recall, which means that all the actual valves were correctly located by the model, and 5% of its predictions were false. In health context, recall usually gets more importance, as it is better to get a false positive than a false negative, i.e., is it better to predict a disease presence in an actual health patient than not identifying the disease [32]. In this case, although the objective is to identify the aortic valve, in the next phase the purpose is to classify this valve as calcified or not, to detect AVC cases. So, between experiment 3 and 4, giving priority to recall, it would be preferable to use rotation technique (experiment 4) for deployment, as it achieved 100% recall.

When including all the images obtained with the four data augmentation techniques, the training time increased significantly, because the dataset size increased images’ processing time, and the results decreased to 91% of precision and 97% of recall. Experiment 4 maintains an advantage over this one. Lastly, training was performed including the two techniques that showed higher results individually – translation and rotation – expecting to get even higher performance. Recall result was 100% again, but precision decreased to 93%, so it has no advantages, when compared to the use of only rotation.

### Aortic valve calcification classification

One approach to automatize diagnosis of Aortic Sclerosis is to use a DL-based classifier that distinguishes between calcified or not calcified valves, in echocardiography images. Like the object detector mentioned in Subsection 3.1., the classifier can both be applied from a pre-trained model using Transfer Learning or be built from scratch. There are many classification models available, such as EfficientNet [33], MobileNet [34] or ResNet [35]. As DL did not show high performance, a heuristic approach was also tested, to compare the results obtained.

#### Classifier modeling

Regarding the development of the DL model for automatic classification, Transfer Learning was used, because it is a great option when having limited labelled data for medical imaging analysis [36], like in the presented context. Various existent models can be used to initialize the network’s weights, and the model must be chosen according to the data to apply it, to get the best performance. Three models were tested - EfficientNet, MobileNet and ResNet.

**Fig. 7:**
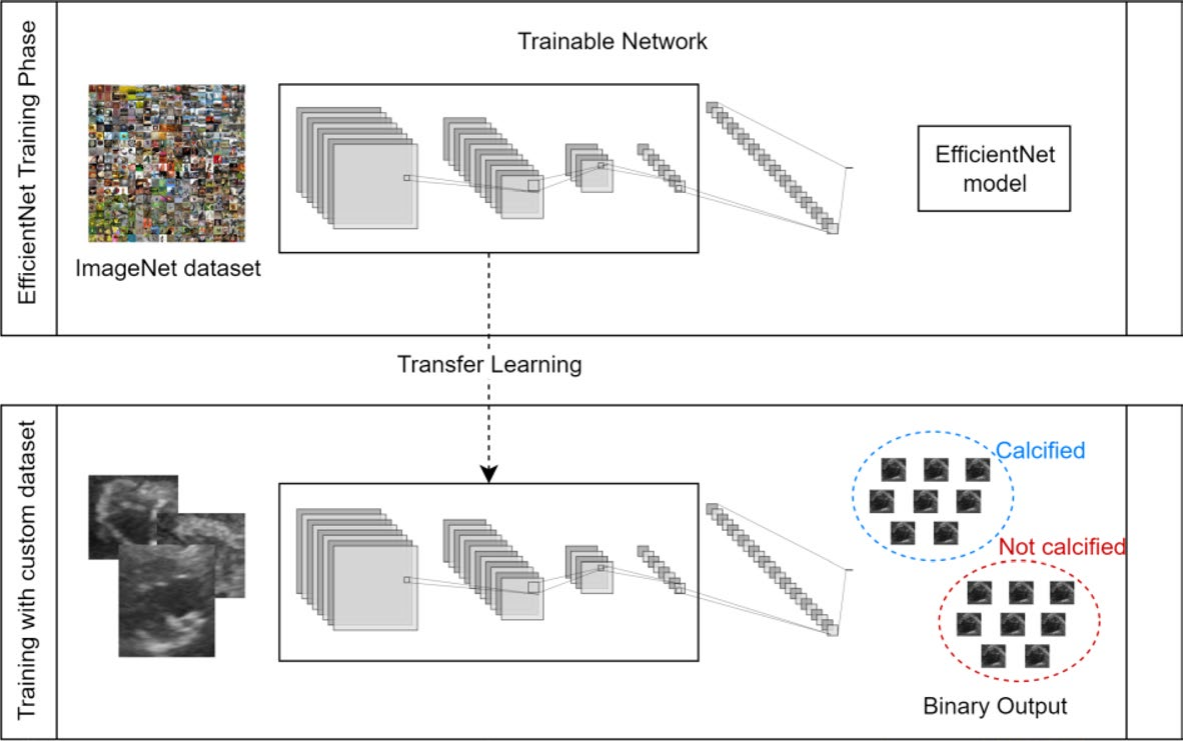
Classification model with Transfer Learning for Aortic Sclerosis classification

Figure 7 represents the use of Transfer Learning with the EfficientNet model, which is similar when applied to other classification models. As can be seen, these models are trained using ImageNet dataset, learning to classify images into categories. When applied to a custom dataset, the first layer will have the pre-trained weights and the next layers are trained with the new data.

#### Experimental procedures

##### Baseline heuristic classification

**Fig. 8:**
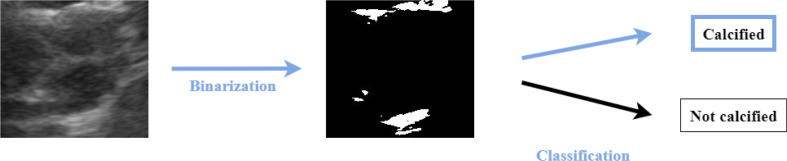
Heuristic classification process

Building upon the semi-automatic approach developed in [9], we adopted a similar methodology for comparative analysis, allowing us to contrast a semi-automatic method with the automatic method proposed in this study. This technique consisted of binarizing images and searching for white pixels (RGB = (255, 255, 255)). If the image has at least one white pixel, the valve is considered calcified. This process is represented in Fig. 8.

The binarization process was manual, to try different thresholds and find the best one. Four different thresholds were tested – 190, 200, 210 and 220 – which means that all the pixels with higher value than the threshold would be set to 255 (white) and the remaining ones would be set to 0 (black). The results obtained for these heuristic approaches are summarized in Table 2.

**Table 2:** Aortic sclerosis classification results for heuristic experiments

Experiment	Dataset size	Accuracy	Precision	Recall	F1-Score	Binarization threshold
1	183	0.42	0.33	0.58	0.42	190
2	183	0.52	0.35	0.39	0.37	200
3	183	0.58	0.40	0.32	0.36	210
4	183	0.39	0.33	0.64	0.43	220

From the four heuristic approaches, the models registered accuracy values between 39% and 58%, precision between 33% and 40% and recall between 32% and 64%, noting that when one of these metrics increases, the other two decreases (for instance, comparing experiments 3 and 4). In this case, as it is preferable to have higher recall than accuracy or precision [37], to minimize the False Negative cases, i.e., AVC cases that are not detected, the best model to choose would be 4, using a binarization threshold of 220. However, this model’s accuracy and precision are quite low, which means that there are many False Positives, i.e., the model identifies too many AVC cases where the patients are healthy.

##### Initial DL experiments

**Fig. 9:**
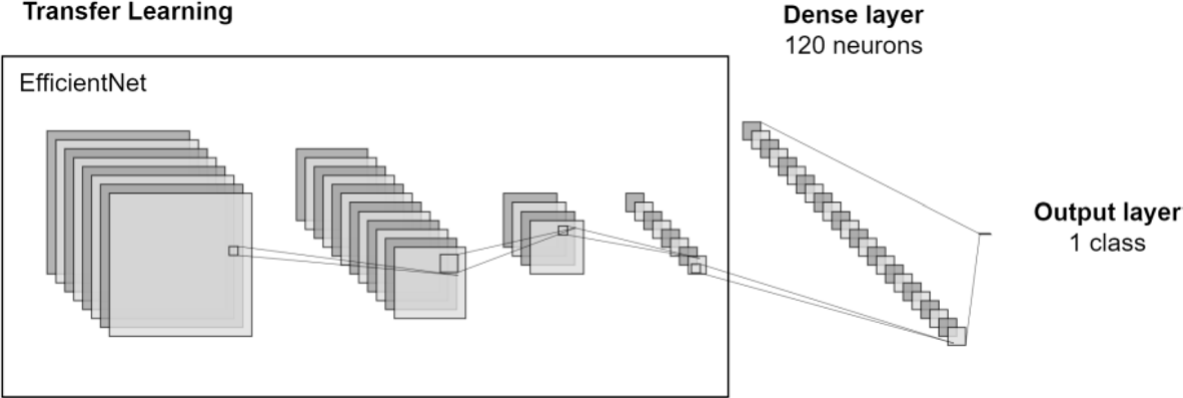
Neural Network architecture

Attempting to get better results, a DL-based approach was followed, consisting of building a Neural Network with the structure shown in Fig. 9. The first layer’s set – responsible for feature extraction and composed by convolutional and pooling layers – uses the pretrained models previously mentioned. The following layer is a dense layer with 120 neurons, and a final output layer performing a binary classification meaning “calcified” or “not calcified”. The model was trained using the full dataset, containing 61 images.

Input shape à (224, 224, 3);

Batch size à 1;

Number of epochs à 50;

Dataset split à 80% for train + 20% for test.

**Fig. 10:**
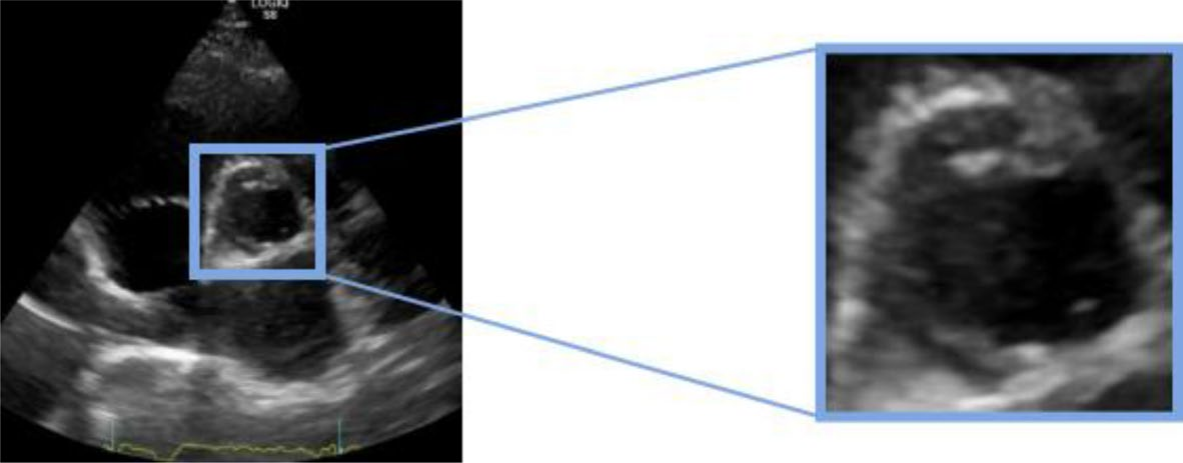
Process of cropping the area of interest

First, the images were entirely used to train the model, i.e., without cropping the valve zone. As result, the model predicted around 50% probability of being calcified, which is a sign that the model did not learn to differentiate between classes. There are studies referring that cropping images can improve the feature extraction process, because when training an image classification model, it tries to extract features from the entire image, which can become less efficient when having useless information [38]. In this case, images were cropped using the original bounding boxes, with a Python script that selected the area between the given coordinates for each image, obtaining the area of interest that contained aortic valve, as illustrated in Fig. 10.

In the next step, for Transfer Learning application, three pre-trained models were used – EfficientNet, MobileNet (and MobileNetV3Small, which is a variation for smaller datasets) and ResNet50. All the three models returned the same output – all the images were classified in the majority class. This means that the network could not extract relevant features from the images, so it predicted the class that was more likely to be correct (the most common).

##### Data pre-processing experiments

**Fig. 11:**
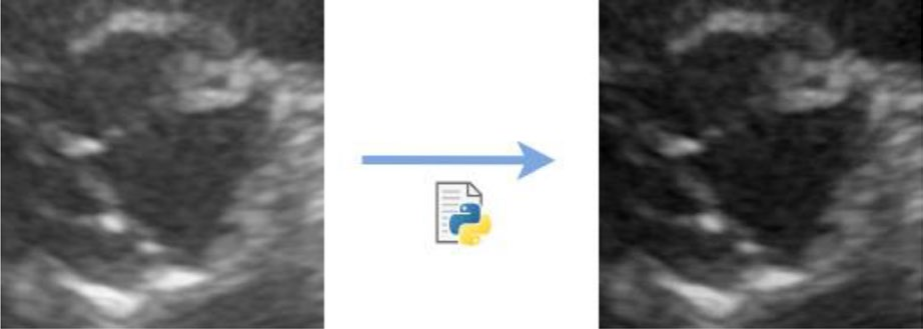
Process of noise reduction

Trying to improve the previous results, the dataset was adapted to avoid biased training of the classifiers. The dataset was modified using the following techniques:

- Noise reduction - Creation of an algorithm that scans all the pixels in an image and subtracts the minimum value to the entire image array. The objective is to turn the darker zones into pure black (RGB = (0, 0, 0)), as demonstrated in Fig. 11.
- Data balancing - The original dataset was unbalanced between the two classes – 22 “calcified” images and 39 “not calcified” images, which can be a problem when the model learns to always predict the same class (the majority one) because the probability of it gets correct is higher [39]. To get around this problem, some images in the minority class were duplicated until the two classes became balanced.
- Data augmentation – Rotations. The rotated images created in data preparation phase, referred to in subsection 2.4., were included to enlarge the dataset. The other types of data augmentation were not adequate to this classification context. Zooms and translations would not work on images containing only the region of interest, and contrast variations would turn the dataset non-uniform.

The following experiments employed two pre-trained models – EfficientNetB0 [40] and MobileNetV3Small [41]. These models were chosen for their simplicity, which is preferrable when working with smaller datasets. More complex models tend to better explain the training data, possibly getting overfitted [42]. The original dataset was used, and two variations were tested – class balancing and data augmentation. The first one equalizes classes distribution and the second one augments the dataset by incorporating rotated images generated during the data preparation phase outlined in Subsection 2.4., were included. The results obtained are presented in Table 3.

In the first experiment, where the original dataset was used for training, both pre-trained models achieved an accuracy of 54%x, which is insufficient to draw conclusions. The second experiment, involving class balancing of the original dataset, yielded 0 TP’s, indicating an inability to correctly classify images with calcified valves, leading to precision and recall values of 0. Thus, this NN is not promising as it fails the identification of unhealthy patients.

**Table 3:** Aortic sclerosis classification results for experiments using different datasets

Experiment	Dataset	Dataset size		Pre-trained model	Accuracy	Precision	Recall	F1-Score
1	Original	61		EfficientNetB0	0.54	0.43	0.60	0.50
				MobileNetV3Small	0.54	0.33	0.20	0.25
2	Balanced	78		EfficientNetB0	0.31	0	0	-
				MobileNetV3Small	0.44	0	0	-
3	With data augmentation	183		EfficientNetB0	0.57	1.00	0.06	0.11
				MobileNetV3Small	0.84	0.92	0.71	0.80
4	Balanced w/ data augmentation	234		EfficientNetB0	0.96	0.96	0.96	0.96
				MobileNetV3Small	0.94	0.96	0.92	0.94

In experiment 3, the performance improved using a larger dataset, rotated images were included without balancing the dataset. EfficientNetB0 returned inconclusive results, displaying 100% precision but only a 6% recall, suggesting a failure to identify the majority of truly calcified valves. In contrast, MobileNetV3Small could achieve more than 70% for all performance metrics, indicating better feature identification and generalization.

When balancing the augmented dataset, NN were trained with 234 images split into two balanced classes. Both pre-trained models had similar performances, achieving over 90% for all metrics. While EfficientNetB0 had consistently 96% for accuracy, precision and recall, MobileNetV3Small showed better precision than recall, remaining more unhealthy patients to be identified.

Experiment 4, yielding the most promising results, highlights the significance of class balancing and data augmentation to enhance model’s performance. Subsequently, this dataset was used to train a NN, with variations in the pre-trained models used for Transfer Learning. Six pre-trained networks – EfficientNetB0, EfficientNetB4, ResNet152V2, ResNet50V2, MobileNetV3Small and MobileNetV3Large – were trained, and the pooling parameter was adjusted to three values for each model – “None”, “Max” and “Avg”. Pooling parameter defines the feature extraction mode during Transfer Learning. When set to “None”, the model’s output mirrors that of the last convolutional layer, while “Max” and “Avg” apply maximum and average pooling to the last convolutional layer, respectively. All the experiments are summarized in Table 4.

**Table 4:** Aortic sclerosis classification results for balanced and augmented dataset using different models

Experiment	Pre-trained model	Map pooling	Accuracy	Precision	Recall	F1-Score
1	**EfficientNetB0**	**None**	**0.96**	**0.96**	**0.96**	**0.96**
2		Max	0.75	0.67	0.92	0.79
3		Avg	0.88	0.88	0.88	0.88
4	EfficientNetB4	None	0.92	1.00	0.83	0.91
5		Max	0.86	0.90	0.79	0.84
6		Avg	0.79	0.89	0.67	0.76
7	ResNet152V2	None	0.85	0.84	0.88	0.86
8		Max	0.77	0.76	0.79	0.78
10		Avg	0.85	1.00	0.71	0.83
11	ResNet50V2	None	0.71	0.63	1.00	0.77
12		Max	0.81	0.8	0.83	0.82
13		Avg	0.77	0.88	0.63	0.73
14	MobileNetV3Small	None	0.94	0.96	0.92	0.94
15		Max	0.69	0.74	0.58	0.65
16		Avg	0.85	0.9	0.79	0.84
**17**	**MobileNetV3Large**	**None**	**0.96**	**0.92**	**1.00**	**0.96**
18		Max	0.77	0.81	0.71	0.76
19		Avg	0.79	0.94	0.63	0.75

For the majority of pre-trained models tested, setting the pooling parameter to “None” achieved better performance. This observation may be attributed to the model’s challenges in feature extraction, particularly given the dataset’s size. Training with a small dataset could limit the model’s ability to extract information from images, and pooling application might further reduce available information.

Among the six pre-trained models used, EfficientNetB0 and MobileNetV3Large stand out with better performances. The first one achieved 96% for all the metrics, while the second one achieved a F1-score of 96%, with a 100% recall, indicating that all the unhealthy patients were identified. In general, simpler NNs demonstrated better performance, likely due to their training with a smaller dataset, requiring less complexity. In this context, considering the importance of recall, MobileNetV3Large without the use of map pooling may be considered the best approach.

## Discussion

To identify Aortic Sclerosis in echocardiography images, the study focused on two main goals: (1) detecting the aortic valve and (2) identifying the presence of calcium in the aortic valve.

In the initial phase, an object detector was constructed to locate the aortic valve in echocardiograms. Transfer Learning was employed from a pre-trained model due to the limited amount of available data. YOLOv5s was chosen as the pre-trained model for its reliability, particularly in handling small datasets.

However, the initial results using the basic dataset were not promising. To address this, various dataset splits were tested using a K-fold Cross Validation method. This analysis revealed that certain darker or noisier images were significantly impacting the overall performance results.

Supposing that 61 images might not be enough for drawing solid conclusions, four types of data augmentation techniques were explored. All of these techniques led to improvements in performance metrics, with two techniques – translation and rotation – standing out by achieving a 98% F1-score.

In the context of health, a higher recall is generally more desirable than higher precision. Recall represents the rate of correctly identified real positives. A higher recall means fewer False Negatives (unhealthy patients) go undetected.

Between the two highlighted data augmentation techniques, rotation would be preferable, since the application of this technique resulted in a 100% recall, surpassing the 95% obtained with translation.

In the second phase, the identification of calcium presence in the aortic valve involved two distinct approaches: (1) a baseline heuristic method without Deep Learning (DL), and (2) a DL-based method using Neural Networks (NNs).

The heuristic approach employed a manual method to binarize images and classify them based on the presence of white pixels. As expected, this method produced low and inconsistent results, failing to provide solid conclusions.

Expecting better performance, a DL-based method was implemented, employing a NN with Transfer Learning from pre-trained models – as used for the object detector in the first phase. Initially, the original images, containing the entire heart, were used to train the model. However, the model’s performance was around 50%. To enhance performance, images were subsequently cropped based on the original bounding boxes limits to focus on the region of interest containing the aortic valve.

Two pre-trained models were selected to be applied – EfficientNetB0 and MobileNetV3Small – because of their simpler architectures, designed for small datasets. When trained with the original dataset, the results were not promising. Trying to improve the model’s performance, two data treatment techniques were tested – balancing and data augmentation. Data augmentation consisted of rotations of the cropped images, and improved the results significantly, when using MobileNetV3Small pre-trained model. When combined with data balancing (making sure each class has a similar number of examples) the results got even higher, for both pre-trained models.

Since data balancing and augmentation resulted in promising results, this dataset was used to test different pre-trained models, for comparison. From six different pre-trained models applied, EfficientNetB0 and MobileNetV3Large resulted in the best performance, with 96% f1-score. These models are known for their simpler structure, which might have resulted in this case with a small dataset. MobileNetV3Large stood out by achieving 100% recall, and since high recall is important for identifying all cases of AVC and preventing undiagnosed cases, it was considered the best model in this context.

## Conclusions

The main purpose of this study was to create an automatic method for detection of Aortic Valve Calcification using DL applied to echocardiograms. To achieve that, it was necessary to (1) create an object detector for aortic valve identification, and (2) to develop an automatic classifier that identified the presence of calcium – an indicator of Aortic Valve Disease.

Data selection was an essential process to obtain the necessary data for applying DL models and achieving the proposed goal. The data collection was conducted in compliance with confidentiality rules, in collaboration with an outpatient clinic. Through anonymization, cleansing, and uniformization processes, a dataset containing 61 echocardiography images – all captured from the same heart view – was compiled for use in the modeling phase. Recognizing that this quantity of data might not be enough to train DL models and achieve satisfactory results, four data augmentation techniques – zooming, translation, rotation, and gamma contrast adjustments – were employed to generate additional images through transformations of the originals.

The final goal was to compile these two algorithms into an automated framework capable of recognizing the aortic valve in an echocardiogram and subsequently classifying the output, thereby indicating the presence of Aortic Sclerosis. Such an advancement in medical workflow would simplify such tasks. Given the limited developments in this field, particularly in the context of echocardiography, as discerned from the conducted literature review, this would indeed represent an innovative advancement.

The object detection model, which aimed to automate the detection of the aortic valve, surpassed expectations, even with a relatively small dataset. The most successful model was built using Transfer Learning from the YOLOv5s network. To train this model, the originally collected dataset was subject to several data pre-processing procedures which involved standardizing the images and implementing data augmentation to expand the dataset. This augmentation consisted of rotating all the images using two small angular variations each, resulting in a dataset three times the original size. This model demonstrated a 95% precision in detecting the aortic valve and achieved a 100% recall rate, meaning that all aortic valves were accurately located by the model in the echocardiography images.

In the subsequent phase, dedicated to developing an automated AVC classifier, the results were also very promising. The best approaches were based on DL, employing Transfer Learning from EfficientNetB0 and MobileNetV3Large pre-trained models – both with a simpler NN architecture. The dataset was pre-processed to address class imbalances, and rotated images were included as an additional data augmentation method to enrich the training dataset. Both experiments yielded F1-scores of 96%. MobileNetV3Large, in particular, stands out by reaching 100% of recall, demonstrating its capability to identify all the Aortic Sclerosis cases.

Throughout the process, the guidance of medical professionals played an essential role, ensuring that the results were aligned with practical objectives. This collaborative effort allowed for continuous adaptation of experiments, aiming to develop a viable solution that aligns with the proposed goal. As a result of these validation steps, it was determined that the Neural Network built for object detection performs a solution for aortic valve identification, improving medical procedures. In contrast, the obtained classifier requires further refinement to achieve higher levels of accuracy and establish itself as a reliable tool for diagnosing Aortic Sclerosis.

## Future work

Overall, the performance of the solutions obtained proved to be positive. However, there is room for further experimentation in the future, and continued development may yield better results. Both the object detection and classification solutions were built with specific parameters that can be adjusted to explore other variations, such as different network architectures or training settings.

The primary challenge encountered through this work was related to data. Acquiring the necessary data requires consistent communication with the clinic and special attention to compliance procedures, making it a time-intensive process. Subsequently, the dataset resulted in 61 images, which can be considered limited when compared to the typically larger datasets required to effectively train Neural Networks and achieve great results. Notably, the issue of limited data was apparently mitigated in the object detection model through the implementation of data augmentation, which did not yield the same level of improvement in the classification model.

Nevertheless, with the anticipation of receiving a greater volume of images in the future, we acknowledge that self-supervised learning will play an essential role in labeling new images that might come unlabeled due to the amount and the manual effort that would take. Furthermore, the inclusion of a classification head during model training will be considered to assess if it enhances the model’s predictive capabilities.

To address this data quantity challenge, it is crucial to ensure that larger quantities of data are available for training these Neural Networks in future developments. Sufficient data enables the model to achieve a deeper understanding of the dataset and extract vital features. Consequently, the model becomes more adaptable to new data and has the potential to achieve higher performance levels.

AVS diagnosis is steadily increasing over the last decade and will need population wide screening and follow up during the years of progression from moderate disease to severe disease requiring intervention, either surgical or percutaneous. The tool developed in this work can be perfected to allow automated screening of population at risk to develop AVS and incrementally proportionate the possibility to follow up patients, without radiation exposure, over the years. With sufficient data, we can explore deeper into understanding the dataset and extract vital features. Namely, we propose moving beyond the binary output (healthy vs. calcified) and exploring the different stages of AVC. This approach could provide valuable insights for clinical diagnosis and treatment planning. The currently proved therapies for AVS are limited to valve replacement at the end stage of the disease. However ongoing research has focused on pharmacologic therapies that can interfere with Aortic Valve Calcification disease progression. Building on the results from this paper it will be possible to test and quantify, noninvasively and without radiation exposure, the effects of such medical interventions.

Furthermore, there are well known risk factors for AVC, such as Hypertension, Dyslipidemia or Diabetes, but the influence of risk factor control on AVC progression is lacking. Our approach raises the possibility of large-scale population studies to evaluate such interventions.

## Data Availability

No datasets were generated or analysed during the current study.
